# Haemolytic anaemia secondary to arsenic poisoning: a case report

**DOI:** 10.4076/1757-1626-2-7768

**Published:** 2009-08-11

**Authors:** Nuno Correia, Catarina Carvalho, Fernando Friões, José P Araújo, Jorge Almeida, Ana Azevedo

**Affiliations:** 1 Department of Internal Medicine, Hospital São JoãoAlameda Prof. Hernâni Monteiro, 4200 - 319 PortoPortugal; 2 Department of Nephrology, Hospital São JoãoAlameda Prof. Hernâni Monteiro, 4200 - 319 PortoPortugal; 3 Intermediate Care Unit, Department of Internal Medicine, Hospital São JoãoAlameda Prof. Hernâni Monteiro, 4200 - 319 PortoPortugal; 4 Department of Hygiene and Epidemiology, University of Porto Medical SchoolAlameda Prof. Hernâni Monteiro, 4200 - 319 PortoPortugal

## Abstract

We report the case of a 56-year-old white man who presented at the Emergency Department for evaluation of dark-red urine. Rapid development of acute renal failure and haemolytic anaemia initially elicited the hypothesis of a haemolytic-uremic syndrome. A previous exposure to a gas mixture containing arsenic and copper was later recognized as the probable aetiology while other differential diagnoses were excluded. Chelating treatment was promptly initiated before laboratorial confirmation of arsenic and copper poisoning. Renal and haematological recovery was gradually observed and the patient survived with no sequelae.

## Introduction

Haemolytic anaemia may be secondary to a wide range of causes either inherited or acquired. The former include disturbances affecting haemoglobin, the membrane-cytoskeleton complex and enzyme deficiency states. The latter include mechanical destruction, drugs, infections, auto-immune disorders or toxic agents.

Toxic agents are an uncommon cause of haemolysis, which may develop even in people without metabolic abnormalities. Many chemicals, due to their oxidative potential, can lead to haemolysis. Other agents act through non oxidative, largely unknown mechanisms, such as arsine, stibine, copper, and lead [1].

Acute poisoning with heavy metals is a rare cause for evaluation in the Emergency Department. Its identification may prove difficult particularly in unconscious patients with no obvious history. A low-level exposure may even be disregarded by the patient himself. This may challenge the initial approach for a problem which can result in a lethal outcome.

## Case presentation

A 56-year-old white man was referred to our Emergency Department (ED) for evaluation of dark-red urine.

The patient complained of a general feeling of sickness, diffuse muscle pain, transient episodes of diaphoresis and chills, with no fever, associated with nausea and bilious vomiting for the past 24 hours. The appearance of dark-red coloured urine, resembling blood, motivated his search for medical care.

He denied abdominal pain or any recent traumatic event. His past medical history was positive for chronic gastritis. He had no past of nephrolithiasis or haematological disorders. He was taking a protein-pump inhibitor and denied use of any over-the-counter substances. He was an occasional pipe smoker and had no history of alcohol abuse or illicit drug consumption. His family history was unremarkable.

On physical examination, his blood pressure was 132/78 mmHg, with a heart rate of 78 beats per minute, a tympanic temperature of 36.8ºC, and a respiratory rate of 18 breaths per minute. There were no signs of dehydration. He had no costovertebral angle tenderness. He had no chronic liver disease stigmata. Further examination, including neurological evaluation, was unremarkable. Initial laboratory data included: Hb = 13.8 g/dL, MCV = 101.4 fL, MCHC = 32.3 g/dL, RDW = 79.8 fl, WBC = 18.01 × 10^9^/L, PLT = 209 × 10^9^/L; CRP = 8.5 mg/L, Cr = 1.55 mg/dL, Urea = 80 mg/dL. Urinalysis revealed proteinuria (3+), leukocyturia (80 cells/HPF), numerous renal tubular epithelial cells, no erythrocyturia, and absent nitrites or urobilinogen. Urinary tract ultrasound excluded signs of lithiasis or obstruction and revealed bilateral renal parenchyma hyperechogenicity and slight perirenal oedema at the right kidney; bladder wall visualization did not show suspicious lesions and the prostate was normal.

He was admitted to the Urology Department for a suspected urinary tract infection. A few hours later his condition deteriorated. He developed jaundice, fever (38.6°C), diarrhoea and mental confusion. His blood panel revealed a normocytic normochromic anaemia with anysocytosis (Hb = 8.0 g/dL, RDW = 83.2 fl), predominantly indirect hyperbilirrubinemia (TB = 5.82 mg/dL, DB = 0.51 mg/dL), high LDH (4415 U/L), elevation of inflammatory markers (WBC = 20.85 × 10^9^/L, CRP = 108 mg/L) and worsening renal dysfunction (Cr = 3.59 mg/dL, Urea = 188 mg/dL) (Table 1).

He was transferred to the Intermediate Care Unit of the Internal Medicine Department. Additional exams included an ECG with normal sinus rhythm and normal QTc interval (416 ms), a normal chest X-ray, a blood smear with rare schizocytes, negative direct and indirect Coombs tests and a fractional excretion of sodium of 3.49%. These data pointed to a haemolytic cause for the rapidly worsening anaemia (Table 1) and acute renal failure in this context.

**Table 1.** In-hospital laboratorial course

	Day 1	Day 1*	Day 2	Day 3	Day 5	Day 6	Day 8	Day 10	Day 18
Hb, g/dL	13.8	11.5	8.0	9.4	8.7	7.9	9.3	9.9	10
WBC × 10^9^/L	18.01	18.44	20.85	10.56	8.71	8.64	12.39	11.82	12.74
PLT × 10^9^/L	209	167	141	69	89	89	124	250	299
CRP, mg/L	8.5	25.4	108.7	52	40.9	28.8	85.5	104	87.9
AST, U/L		112	121			207	57	34	35
ALT, U/L		33	36			193	156	91	31
γGT, U/L		18	19						27
ALP, U/L		71	62						43
TB, mg/dL		2.06	5.82	6.16		0.94	1.02	0.66	0.63
DB, mg/dL		0.43	0.51	0.48		0.23	0.72	0.15	0.14
LDH, U/L			4115	1430		997	659	549	289
Urea, mg/dL	80	135	188	182	144	112	70	65	59
Cr, mg/dL	1.55	2.56	3.59	4.96	4.43	4.34	3.45	3.86	2.79

Exposure to a gas mixture containing arsenic and copper occurred nearly 32 hours before initial medical evaluation. One session of plasmapheresis was performed at in-hospital day 2. Penicillamine was administrated from day 4 to 7, followed by dimercaprol during 10 days. Transfusion with 1 pack of red blood cells was performed at day 2. Haemolytic anaemia, renal dysfunction and hepatotoxicity recovered gradually after chelation therapy.

* Laboratorial results at approximately 12 hours of admission.

The association of acute renal failure and haemolytic anaemia elicited the hypothesis of a haemolytic-uremic syndrome. Considering other causes for a haemolytic physiopathology which could not be immediately excluded and the severity of the clinical condition of the patient, plasmapheresis with fresh frozen plasma was initiated and RBC transfused as needed.

Clinical history was revisited. The patient owned a factory of zippers where chemical compounds were frequently used in plating techniques. At this time the patient recalled accidental inhalation of a possible toxic gas nearly 32 hours before, while performing a new plating process wearing no protection. The gas resulted from the mixture of a so-called “tin’s oxidant” with a metal alloy. This exposure lasted no longer than 5 minutes and nearly 4 hours later he remembered feeling “sick”. Detailed information about this chemical substance was immediately requested.

At day 3, nearly 90 hours after the accidental exposure, the composition of the toxic substance was known. “Tin’s oxidant”, as the patient named it, included 40-45% of chlorhydric acid (HCl), 1.2-1.4% of copper sulphate (CuSO_4_), and 5-6% of arsenic trioxide (As_2_O_3_). Plasmapheresis was stopped at this time.

Blood and 24-hour urine samples were sent to the laboratory for measurement of arsenic and copper levels. Chelation treatment was initiated with Penicillamin (1800mg/day *per os*), before laboratorial confirmation, in order to address both copper and arsenic intoxication. Initial therapy with intramuscular Dimercaprol was postponed due to thrombocytopenia.

The patient completed 3 days of Penicillamin, followed by the administration of intramuscular Dimercaprol 200 mg at the following regime: 4-hourly in the first 6 days, then 6-hourly at day 7 and 8, and 12-hourly at day 9 and 10. Simultaneously, forced alkalinisation was performed, with fluid therapy as necessary.

**Figure 1.**
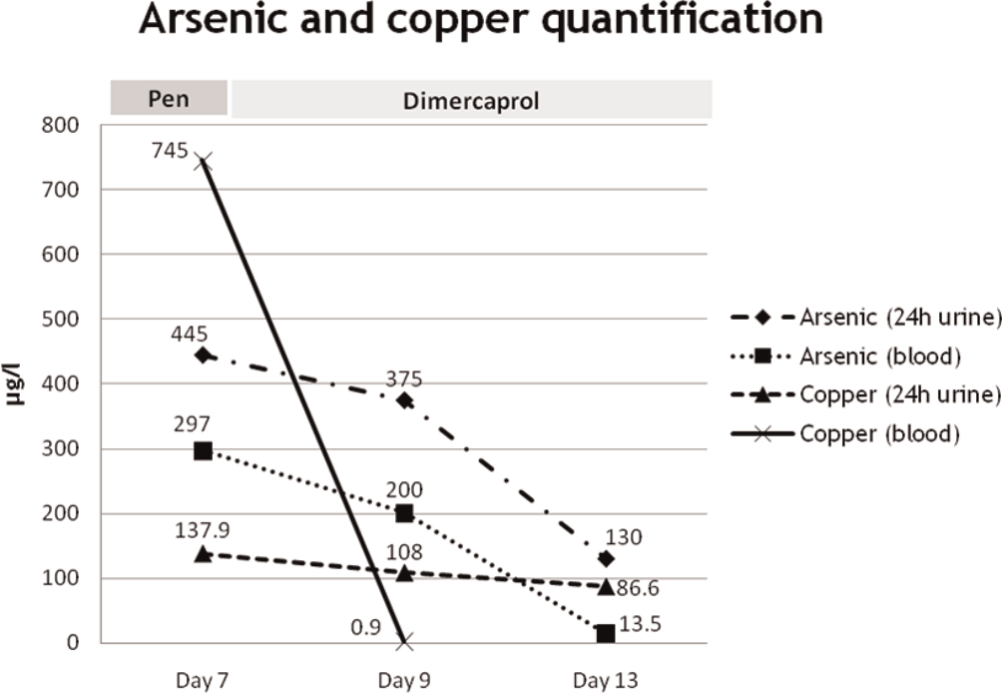
Plotted concentrations of arsenic and copper in blood and urine samples under chelation therapy. Chelating therapy was preceded, three days before, by one session of plasmapheresis. Penicillamine (Pen) was administrated between days 4 and 7, followed by dimercaprol from days 8 to 18. *Normal values*: arsenic (24 h urine) <100 µg/L; arsenic (blood) <70 µg/L; copper (24 h urine) = 15-60 µg/L; copper (blood) = 0.7-1.5 µg/L.

Quantitative measurements were available at in-hospital day 7 and confirmed high levels of arsenic and copper in urine and blood (Table 2). Further measurements demonstrated a progressive decline under the expected chelation reaction (Figure 1). In repeated blood gas analysis there was no methaemoglobin, a possible consequence of copper intoxication.

**Table 2.** Arsenic and copper blood and urine concentration 90 hours after exposure (available at in-hospital day 7)

	Results (µg/L)	Normal values
Arsenic		
24h urine	445	<100 µg/L
Blood	297	<70 µg/L
Copper		
24h urine	137.9	15-60 µg/L
Blood	745	0.7-1.5 µg/L

During in-hospital stay, patient’s symptoms gradually vanished and recovery of haematological, renal and liver disturbances was observed. He did not present any neurological or respiratory abnormalities. Syalorrhea and inflammatory signs at intramuscular injection sites developed as a consequence of dimercaprol treatment. After 13 days of chelation he was discharged and referred for follow-up appointments at the Internal Medicine and Nephrology Outpatient Clinic.

One month later he remained asymptomatic. Physical examination was unremarkable. Six months later his haemoglobin level and renal function were normal (Hb = 14.8 g/dL; MCV = 89.1 fL; MCHC = 34.1 g/dL; Cr = 1.02 mg/dL, Urea = 46 mg/dL). The patient was discharged.

## Discussion

Arsenic is one the four most hazardous toxicants, along with cadmium, lead, and mercury. Metal poisoning can result from exposure to inhaled dusts, fumes or vapours. Another possible route comes from ingestion of contaminated food, drinks or by hand-to-mouth exposure [2-4].

This patient presented with many of the classical clinical findings attributed to arsenic acute intoxication. Initial unspecific features of acute gastrointestinal findings may be underappreciated, particularly if a recent exposure is not known. Acute toxicity with arsenic manifests within hours as nausea, vomiting, diarrhea, abdominal pain. Severe poisoning may result in multi-organic failure, delirium, seizures, coma, and ultimately death. If the patient survives, bone marrow suppression, peripheral neuropathy and skin lesions may develop [2-4].

Haemolytic anemia secondary to acute intoxication with arsenic is a well-recognized effect [2-4]. Arsenic-related haemolysis may also be a consequence of chronic intoxication [5].

The gaseous state is one the most toxic forms of arsenic [6]. Cases of intoxication with arsine gas have been published mostly as a result of accidental work-related exposures [6-9]. Because arsine is nonirritating and produces no immediate symptoms, persons exposed to hazardous levels may be unaware of its presence. In contrast with reported cases following arsenic poisoning [8,10-17], our patient did not develop respiratory failure.

Additionally, direct heart toxicity may result in disturbances of the electrical conduction system [2-4,15]. One report described an early manifestation of *torsade* *de pointes* after acute arsenic poisoning [18].

We can only retrospectively speculate about the initial exposure dosage, which possibly was not high enough to implicate direct respiratory or cardiac damage.

Treatment should be promptly initiated under high suspicion of a recent intoxication. Primary treatment in acute arsenic poisoning involves rapid decontamination and early initiation of chelation therapy with agents such as dimercaprol (British Anti-Lewsite, BAL), edentate (EDTA), succimer (DMSA, dimercaptosuccinic acid) and penicillamine. No specific antidote is available and there are no controlled trials regarding different therapeutic strategies [2-4].

A recent case report has suggested plasma exchange as an effective early treatment intervention for acute arsenic poisoning [19]. In our case, plasmapheresis was performed regarding the hypothesis of a haemolytic-uremic syndrome, before any toxicodrome was suspected. However, it may have contributed for the benign clinical course since chelating treatment was only initiated nearly 100 hours after exposure.

In this case, it is not possible to separate the haemolytic effect of copper from the arsenic one, since copper intoxication may also cause haemolysis [20]. Methaemoglobinemia is a frequent complication of copper poisoning which may require additional therapeutic strategies [20]. Its absence in the presented case points against a major contribution of this metal in patient’s clinical course.

## Conclusion

We report a rare case of haemolytic anaemia following arsenic and copper acute intoxication. The unusual nature of this aetiology and the delayed awareness of a possible toxic exposure challenged the diagnosis.

Common consequences of arsenic poisoning are haemolysis and renal failure.

Because of the severity of the clinical course, including multi-organic failure and a high risk of death, therapeutic strategies should presumptively be initiated without formal laboratory confirmation. Evidence-based treatment options are limited. Use of chelating agents, such as dimercarprol, is recommended. Whether plasma exchange should be a primary, an alternative or additional treatment option remains unclear, although its early use may be an effective treatment.

## Abbreviations

ALP: alkaline phosphatase
Cr: creatinine
CRP: C-reactive protein
DB: direct bilirubin
FENa: fractional excretion of sodium
γGT: gamma glutamyl transpeptidase
Hb: haemoglobin
HPF: high power field
LDH: lactate dehydrogenase
MCHC: mean corpuscular haemoglobin concentration
MCV: medium corpuscular volume
PLT: platelet
RDW: red cell distribution width
TB: total bilirubin
WBC: white blood cell
